# An Updated Perspective on the Dual-Track Model of Enterocyte Fat Metabolism

**DOI:** 10.1016/j.jlr.2022.100278

**Published:** 2022-09-10

**Authors:** Joshua R. Cook, Alison B. Kohan, Rebecca A. Haeusler

**Affiliations:** 1 Naomi Berrie Diabetes Center, Columbia University College of Physicians and Surgeons, New York, NY, USA; 2 Division of Endocrinology, Diabetes & Metabolism, Department of Medicine, Columbia University College of Physicians and Surgeons, New York, NY, USA; 3 Division of Endocrinology and Metabolism, Department of Medicine, University of Pittsburgh, Pittsburgh, PA, USA; 4 Department of Pathology and Cell Biology; Columbia University College of Physicians and Surgeons, New York, NY, USA

**Keywords:** intestine, lipid absorption, lipid storage, lipid transport, apical, basolateral, enterocyte, triglyceride, chylomicron, aCLD, apical CLD, bCLD, basolateral-track CLD, CLD, cytoplasmic lipid droplet, DNL, de novo lipogenesis, eDNL, Enterocyte de novo lipogenesis, FA, fatty acid, FAO, fatty acid oxidation, FFA, free fatty acid, GLP-1, glucagon-like peptide-1, SME, second-meal effect, TG, triglyceride, TRL, triglyceride-rich lipoprotein

## Abstract

The small intestinal epithelium has classically been envisioned as a conduit for nutrient absorption, but appreciation is growing for a larger and more dynamic role for enterocytes in lipid metabolism. Considerable gaps remain in our knowledge of this physiology, but it appears that the enterocyte’s structural polarization dictates its behavior in fat partitioning, treating fat differently based on its absorption across the apical versus the basolateral membrane. In this review, we synthesize existing data and thought on this dual-track model of enterocyte fat metabolism through the lens of human integrative physiology. The apical track includes the canonical pathway of dietary lipid absorption across the apical brush-border membrane, leading to packaging and secretion of those lipids as chylomicrons. However, this track also reserves a portion of dietary lipid within cytoplasmic lipid droplets for later uses, including the “second-meal effect,” which remains poorly understood. At the same time, the enterocyte takes up circulating fats across the basolateral membrane by mechanisms that may include receptor-mediated import of triglyceride-rich lipoproteins or their remnants, local hydrolysis and internalization of free fatty acids, or enterocyte de novo lipogenesis using basolaterally absorbed substrates. The ultimate destinations of basolateral-track fat may include fatty acid oxidation, structural lipid synthesis, storage in cytoplasmic lipid droplets, or ultimate resecretion, although the regulation and purposes of this basolateral track remain mysterious. We propose that the enterocyte integrates lipid flux along both of these tracks in order to calibrate its overall program of lipid metabolism.

## Preface

In recent decades it has become apparent that intestinal fat handling extends beyond the purely absorptive. For one, the intestine senses and responds to bioactive signaling lipids, derived from the diet or the commensal microbes, but even as it pertains to bulk lipid homeostasis, we can now consider multiple facets of the enterocyte’s relationship with fat and how this intercalates into metabolism writ large. We therefore intend not to present an exhaustive recapitulation of the literature on this topic. Rather, we will synthesize the themes that emerge and analyze their potential connections to human health and disease. We focus on human data while using work from model systems to bridge knowledge gaps, and in both cases, we emphasize studies that are physiologically integrative. For expert review of the mechanistic details of intestinal lipid uptake, deposition, and remobilization, we refer the reader to multiple recent publications (1, 2, 3, 4, 5, 6, 7).

## Intestinal Reverse Lipid transport and the Dual-Track Hypothesis

The canonical anterograde lipid-absorptive pathways within enterocytes have been extensively studied given their obvious physiologic importance. However, as early as the 1950s, evidence of *reverse* intestinal lipid trafficking, that is, transcellular efflux of cholesterol from the circulation into the intestinal lumen, began to emerge (8, 9, 10, 11, 12). The colocalization of cholesterol and triglycerides (TGs) within lipoproteins naturally raises the question of a parallel pathway for fats, and, in fact, there is general agreement that intestinal epithelial cells take up circulating fat across their basolateral membrane. Yet, unlike cholesterol, circulating fatty acids (FAs) are not known to undergo transintestinal excretion, calling into question the enterocyte’s purpose in taking them up.

**Fig. 1:**
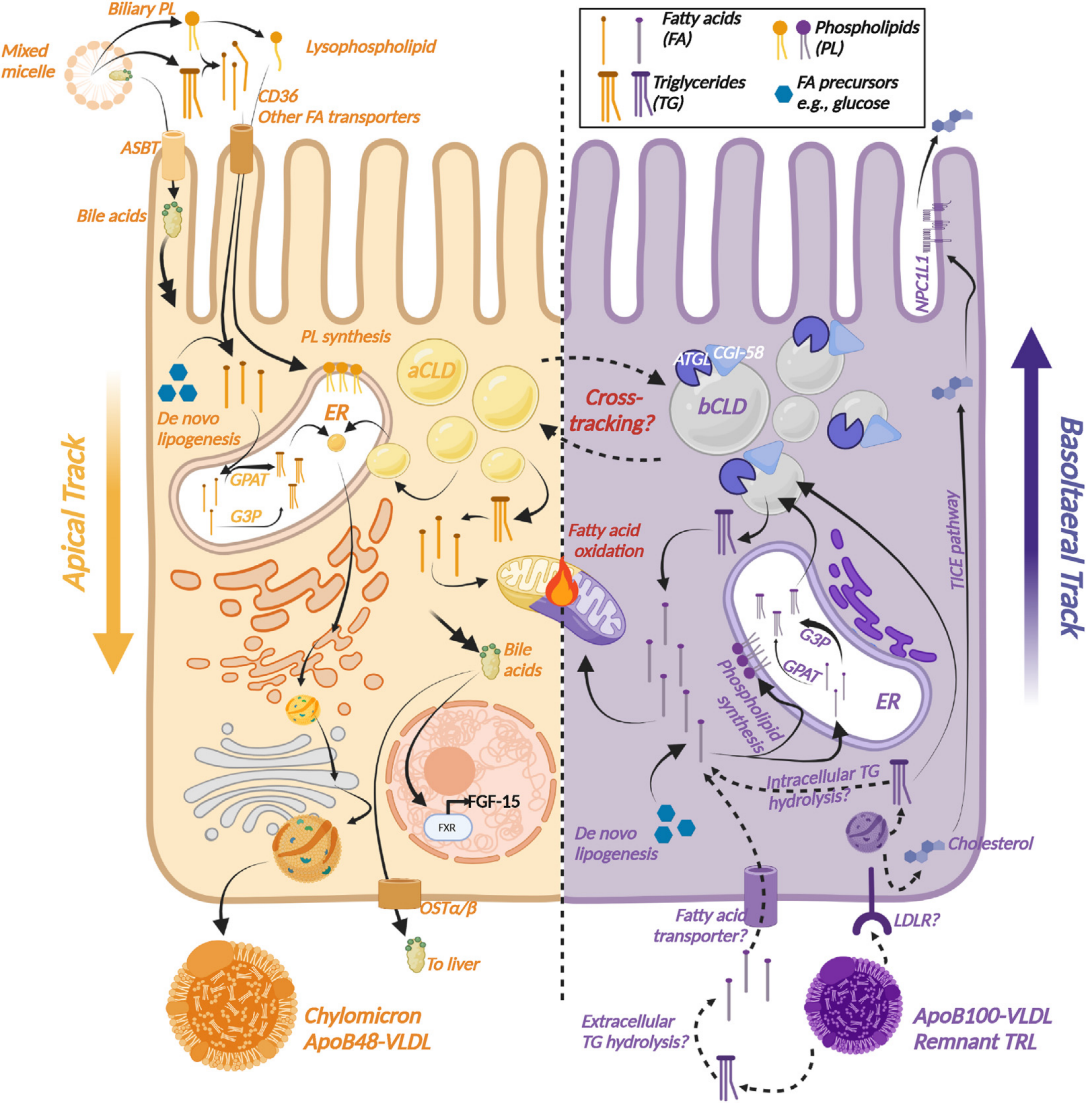
Dual-track model of enterocyte fat metabolism. The apical track is illustrated in yellow on the left side of the schematized enterocyte and the basolateral track on the right, in purple. Both tracks feature uptake of TG- or de novo lipogenesis (DNL)-derived fatty acids that are used to synthesize phospholipids (PLs), oxidized, or reesterified to TGs via the G3P (predominant in basolateral track) and GPAT (predominant in apical track) pathways. Reesterified TGs are stored in cytosolic lipid droplets in both tracks, and although bCLDs express a different complement of CLD-associated proteins than do aCLDs, these droplets or their contents may “cross-track.” The source of basolateral-track fatty acids—via uptake of VLDL or chylomicron remnants for intracellular hydrolysis versus extracellular TRL hydrolysis—remains unclear. Bile acids reabsorbed from the intestinal lumen also regulate enterocyte gene expression patterns before recirculating to the liver. Cholesterol taken up from the circulation is excreted via the basolateral track through the transintestinal cholesterol excretion (TICE) pathway.

**Fig. 2:**
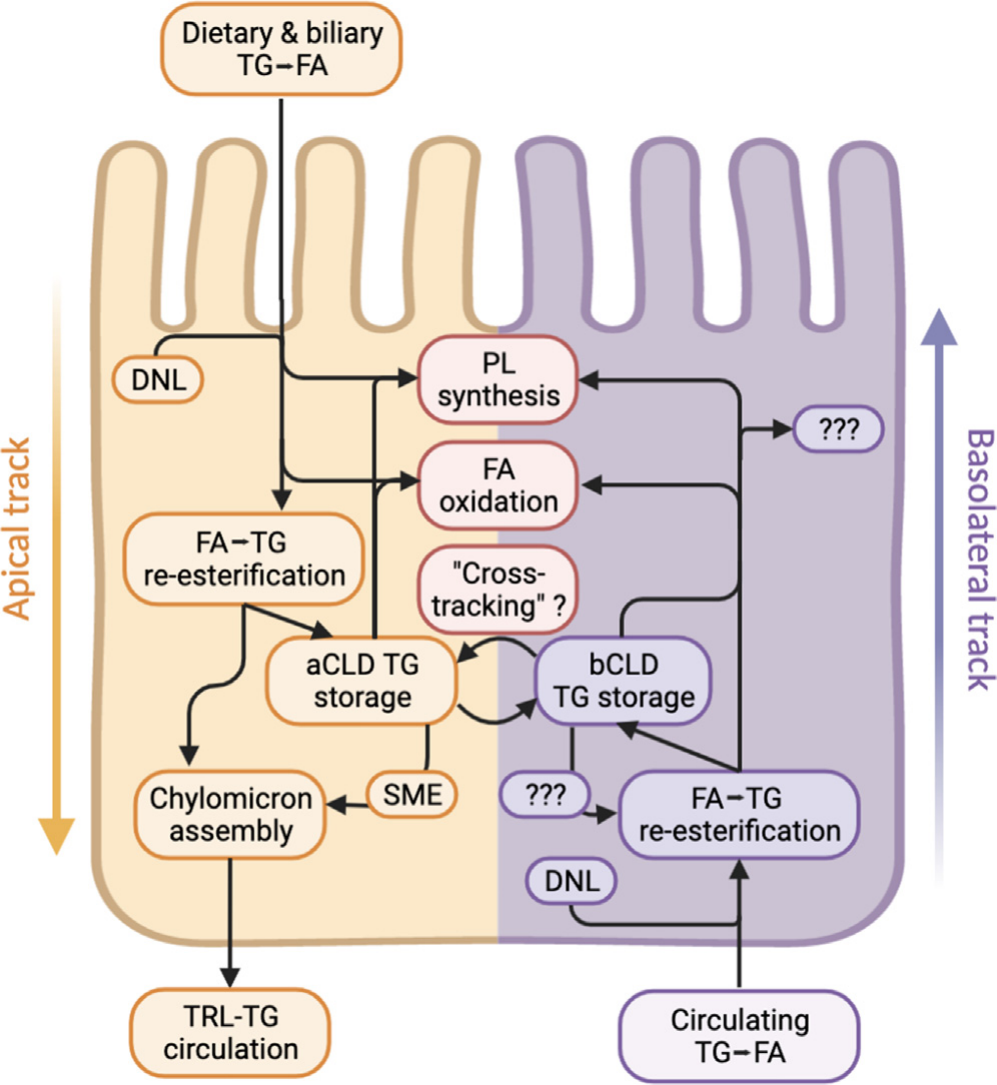
Functional interaction of the dual tracks. Summary of known functions and interplay of the two enterocyte lipid tracks. The apical track, illustrated in yellow on the left side of the schematized enterocyte, demonstrates the absorption and utilization of dietary lipid, including re-esterification for storage in aCLD and/or chylomicron synthesis and secretion, phospholipid (PL) synthesis, and fatty acid (FA) oxidation. Additional apical track lipid is provided by de novo lipogenesis (DNL) from non-lipid precursors (e.g., carbohydrates) and perhaps “cross-tracking” of basal-track fats. These functions appear to be largely mirrored in the basolateral track, illustrated in purple at right, demonstrating a largely similar array of fates for circulating fats following basolateral uptake. However, there remain unknown aspects of basolateral track function remain.

The very existence of substantial basolateral fat uptake and storage by the human enterocyte suggests some degree of participation in *systemic* fat metabolism beyond its canonical role in enteral nutrient absorption and secretion. A wealth of animal-based evidence substantiates this inference (13, 14, 15, 16, 17, 18, 19) and suggests that lipid flux within the enterocyte operates on a dual-track system (13, 15, 16). First, the canonical, or apical, pathway traffics in newly absorbed dietary fat, which may be directly secreted into lacteals as chylomicrons or deposited in dedicated cytoplasmic lipid droplets (CLDs) for later use. Second, the apical track is joined by an ill-defined basolateral counterpart comprising fat taken up from the blood and also stored in CLDs, but whose ultimate metabolic destiny remains largely unknown. Significantly, these two tracks appear to be segregated: lipids absorbed basolaterally seem to commingle little with those that the same cell has absorbed apically, suggesting distinct CLDs and cellular machinery subserving them (13, 15, 16) (Figs. 1 and 2).

## The Apical Track

Although the apical track’s textbook role of importing dietary fat is obvious, closer examination reveals that the enterocyte is not merely a passive absorptive conduit. Rather, the enterocyte deliberately sequesters some of the fat that it brings on board within apical CLDs (aCLDs), implying a more dynamic role in the regulation of lipid metabolism.

### The second-meal effect

Suspicion that the healthy intestine siloes at least a portion of absorbed lipid for later release originated in the repeated observation of a “second-meal effect” (SME), in which one lipid-rich meal apparently triggers an early surge in circulating apoB-48-containing intestinal triglyceride-rich lipoproteins (TRLs) during a second meal that occurs too quickly to be accounted for by the latter (20, 21, 22, 23, 24, 25, 26, 27). More compelling still, circulating chylomicron-TG levels still swiftly rise when the second meal is comprised solely of carbohydrates (i.e., fat free) (22, 28, 29). Stable-isotope tracer studies have demonstrated that the chylomicron-TG appearing after later-meal ingestion may be derived from lipid intake more than 18 h prior (30) and mirror the FA composition of the first meal (21, 22, 24). The discovery of duodenal (31) and jejunal (28) enterocytic CLDs in human intestinal biopsy specimens corroborated the intestinal storage of dietary fat inferred from the second-meal effect. Two SME studies in humans documented fewer (31) and smaller (28, 31) CLDs per cell on biopsy if subjects ingested a carbohydrate load versus water at 5–6 h after a high-fat first meal, suggesting selective CLD depletion to serve the SME. However, biopsies were not taken after an overnight fast or in the immediate first-meal postprandial period to allow for direct comparisons of individuals’ CLD morphology over time (28, 31).

Alternative lipid sources may also contribute to the observed SME. For instance, enterocytes recover FAs derived from biliary phospholipid excretion for esterification to TGs (32) and biliary phospholipids themselves are recycled as chylomicron coating (33, 34). Thus, as duodenal entry even of pure carbohydrate triggers some bile excretion (35, 36), the SME might represent the reappearance of biliary phospholipid-derived FAs in TGs. It is worth noting that only about 50% of bile is stored in the gallbladder during fasting and released with a meal; the other half drains into the duodenum, even during fasting (37). Indeed, during fasting, biliary lipids are the primary sources of lipids in the mesenteric lymph (38). Because glycerolipid hydrolysis reesterification likely proceeds too slowly for phospholipids from postprandial gallbladder emptying to account for the SME (25), SME TG may draw upon biliary lipids absorbed during fasting or a prior meal.

Although the *existence* of the SME is clear in a phenomenological sense, its biological basis and purpose remain hazy. This has been difficult to sort out experimentally as the SME has been difficult to model in mice (13); we present a few hypotheses worth considering. First, early mobilization of reserved dietary fat may “prime the pump” to efficiently ramp up the postprandial chylomicron assembly line (25, 39). This may be particularly important when FA species enzymatically preferred—or perhaps even required—for TG esterification are limiting (40). Another possible explanation for the SME is that constitutive low-level chylomicron production from aCLDs (25, 41) protects other tissues from large TG excursions due to lipoprotein lipase saturation (25), although the SME would not be optimally timed for this purpose. On the other hand, intestinal lipid stockpiling may act as a defense against starvation. If so, then the renewed availability of dietary nutrients would temporarily relieve the intestine of this responsibility. In this vein, the SME may prove an exercise in self-defense if it represents a hurried divestiture of banked TGs in anticipation of a glut of new dietary fat. Because the enterocyte can foresee neither the quantity nor the composition of fat that it will ultimately encounter over the course of the meal, it would be prudent to ensure optimal readiness of its finite machinery to process the incoming lipid load. Once it has averted the threat of acute FA overload, the intestine can then restock its cache with fresh TGs, perhaps to ration as needed during fasting. Note that each of these hypotheses involves a preparatory action on the part of the enterocyte; this would square with SME triggering by “cephalic-phase” nutrient sensing (i.e., based on taste and smell), ostensibly as an early warning system of an impending food bolus (30, 42).

### The apical track beyond the second-meal effect

Whatever its purpose, the SME casts aCLDs as more than just a brief stopover for TGs waiting their turn to be packaged in chylomicrons. As alluded to above, aCLDs allow enterocytic participation in quantitative and qualitative FA balance, a theme likely common to both tracks. Because of inherent enzymatic substrate preferences, the enterocyte scrambles the original FA composition of dietary TGs in their reassembly for chylomicron packaging (40), hence the distinct FA composition of dietary versus circulating TGs after a meal (43, 44, 45). The enterocyte may therefore be able to call upon aCLDs as a clearinghouse to mix and match FAs for esterification as circumstances dictate (45, 46, 47).

We can look as well to the external signals that influence nutrient flux along the apical track for insights into its other roles. Bile acids promote secretion of incretin hormones and other neuropeptides by enteroendocrine cells (48, 49, 50, 51, 52) and fibroblast growth factor-19 (FGF-19) secretion by enterocytes (53). Typifying the former, glucagon-like peptide-1 (GLP-1) receptor agonist administration both to healthy volunteers (54) and to patients with type 2 diabetes (55) acutely diminishes postprandial secretion of apoB-48-containing TRL, while GLP-2 administration does just the opposite (56). Attenuation of TG secretion by GLP-1 may, for example, help to coordinate the systemic after-meal switch to preferential utilization of glucose over fat (57), which in turn could entail temporary sequestration of a portion of dietary TGs in aCLDs. As human enterocytes do not express GLP receptors (56, 58), these observations reflect indirect regulation via some intermediary—perhaps through signals from neighboring cells or secondary to incretin-stimulated insulin secretion.

Alterations of gut hormones may represent only a portion of the lipid-regulatory activities of bile acids in the intestine. Treatment with chenodeoxycholic acid, an endogenous human bile acid species, lowers TGs in hyperlipidemic patients (59, 60), while bile acid-binding resins acutely lower circulating bile acids and raise TGs (61, 62). The molecular mechanism of this well-known observation remains enigmatic (61); it may arise from bile acid regulation of chylomicron secretion (63) and/or from direct or indirect (*cf.* FGF-15/19) bile acid stimulation of hepatic de novo lipogenesis (DNL) (64, 65).

## Laying the Groundwork for a Basolateral Track

### Origins of basolateral-track fats

While dietary lipids are the source of apical track substrates, the source of lipids for the basolateral track is less clear. Before considering the purpose of a basolateral track, it would be useful first to establish that lipids would enter the basolateral track through an actively regulated, singular process in enterocytes. Such possibilities include (re)esterification of basolaterally absorbed free fatty acids (FFAs), basolateral TRL-TG reuptake, or intestinal DNL from circulating carbohydrate precursors. Each of the possibilities is supported by existing data, albeit with varying degrees of directness.

#### (Re)esterification of basolaterally absorbed free FA and glycerol

Basolateral uptake of circulating FAs by enterocytes has been well established in animal models (13, 16, 17, 66, 67). There is also human in vivo evidence of basolateral uptake as an ultimate source of TRL-TG: ^14^C-palmitate infused intravenously was recovered within minutes in jejunal biopsy homogenates (68), and IV-infused deuterated glycerol reappeared within circulating chylomicron-TG (69). Additionally, positron emission tomography imaging demonstrated duodenal and jejunal avidity for ^18^FTHA, a nonoxidizable palmitate analogue, in both lean and, to a greater extent, obese humans (70). These important studies, however, lacked the spatial resolution necessary to localize their findings specifically to enterocytes versus the extraenterocellular lamina propria (71).

The metabolic fate of FFAs within enterocytes may differ by site of uptake, in keeping with the dual-track model. In both animals (16, 17) and humans (68), apical FFAs are mainly esterified to TGs, while the fate of basolateral FFAs has traditionally has been seen primarily as oxidation or incorporation into phospholipids (1). It is not necessarily surprising, however, that basolaterally absorbed FFAs were found to be principally destined for oxidation or structural lipid synthesis, as studies were generally performed in the fasting state. As fasting may impel enterocytes, as other cells, to rely more heavily on β-oxidation of FAs as an energy source, the destiny of basolateral FFAs could coordinately differ under fed conditions (15, 16, 19). Consistent with this possibility, intravenously infused tritiated oleate continued to be taken up by the intestinal mucosa during concomitant enteral administration of glyceryl trioleates in rats; intestinal mucosal specific activity was recovered almost entirely esterified within TGs (15). Only a small proportion of this basolaterally derived TGs was ultimately incorporated into chylomicrons, again consistent with the dual-track hypothesis (15). Although comparable human tissue-level data are not available, intravenous infusion of a lipid emulsion in fed healthy volunteers acutely ramped up apoB-48 production (i.e., chylomicron secretion) without affecting its catabolism (72). Thus, to the extent that this rise in apoB-48 production reflects augmented availability of TGs derived from basolaterally delivered FFAs, the rat data suggest that the enterocyte stows away an even larger share of that newly esterified TGs in basolateral-track CLDs (bCLDs) than it secretes (15, 72). The track-differential routing of FFAs into TG synthesis may be reinforced by distinctive methods of reesterification (73): the apical track primarily utilizes the less common monoacylglycerol pathway (74, 75), while the basolateral track may favor the more widely used glycerol-3-phosphate pathway (16, 73, 76).

#### Basolaterally absorbed lipoprotein-TG

The enterocyte lipid pool may draw upon FA/TG taken up basolaterally from lipoproteins, perhaps in the form of recently secreted chylomicrons or recirculated chylomicron remnants (13, 18, 19). As with other cells in the body, these lipoprotein-derived fats may undergo basolateral absorption as holoparticles entering the endolysosomal system or as FFAs locally derived from extracellular hydrolysis (13, 19). Concordantly, both basolateral uptake of TRL/remnants and the constituent TG’s subsequent resecretion as chylomicrons have been demonstrated in rodent enterocytes (14, 19). Although these events have not yet been reported in humans, human enterocytes do express the LDL receptor on their basolateral surface (77, 78), suggesting the capacity for uptake of apoB100/E-containing TRL. That HDL does not represent a major source of cholesterol for transintestinal cholesterol excretion in mice further implicates basolateral uptake of non-HDL particles by enterocytes as a potential source of TGs (79). Yet, the failure of PCSK9 inhibitor treatment to meaningfully affect postprandial chylomicron-TG levels or apoB-48 secretion in humans argues against a central role for enterocyte LDLR in this process (80, 81, 82).

#### Enterocyte de novo lipogenesis

Enterocytes can also synthesize TGs from precursor substrates, and we surmise this includes those precursors taken up basolaterally from the circulation. Pure carbohydrate consumption increases circulating apoB-48 and chylomicron-TG levels in human volunteers, suggestive of human enterocyte DNL (83, 84, 85). Enterocyte de novo lipogenesis (eDNL) with coordinately increased chylomicron production has been conclusively demonstrated in rodent enterocytes (13, 86, 87), while human enterocytes at a minimum do show mRNA expression of the full suite of required enzymes, including acetyl coA carboxylase and FA synthase (88). Active DNL has not yet been specifically demonstrated in human enterocytes, but human duodenal explants do exhibit wholesale DNL (89), and in vivo stable-isotope tracer studies have documented incorporation of moieties derived from enteral fructose (90, 91) and from intravenous glycerol (92) in chylomicron-palmitate. The additional time required for eDNL from carbohydrate delays the secretion of resultant chylomicron-FA/TG and apoB-48, represented as a shoulder or second peak on the curve, after the absorption of the dietary lipid component of the original meal. Interestingly, the second peaks of apoB-48 and chylomicron-lipids coincide with, or even follow, the postprandial rise in hepatic apoB-100 and VLDL secretion (84, 85, 90, 91), and rates of hepatic and intestinal DNL appear to be very tightly correlated (92). These observations leave open the possibility, mentioned above, that at least some of the second rise in chylomicron secretion attributed to eDNL may actually reflect basolateral uptake and reprocessing of recirculated, hepatically derived lipids.

### Distinguishing basolateral CLDs

If the basolateral track does contribute to the enterocyte lipid pool by any or all of the above mechanisms, dedicated bCLD could, speculatively, enable spatiotemporal control over enterocyte lipid-metabolic processes. This, however, would require recognition and differential regulation of two coexisting CLD populations: aCLD versus bCLD. Mouse duodenal enterocytes have been found to contain CLDs whose lipid makeup clearly differs from those in other cell types (47). Enterocyte CLDs also feature an adipocyte-like complement of lipid droplet-associated proteins (93, 94) that may further distinguish bCLD from apical (13, 47). Mouse enterocyte-specific knockout of two major CLD-associated proteins, adipose TG lipase and comparative gene identification-58 (CGI-58), results in massive enteral steatosis despite untrammeled dietary fat absorption and chylomicron secretion (13). The selective enterocytic accumulation of intravenously administered FAs, on the other hand, implies that the missing proteins specifically regulate bCLD (13, 95), consistent with a unique bCLD identity.

## Exploring the Significance of the Basolateral Track

### Housekeeping functions

If we accept the above observations as evidence that basolateral track represents a discrete, organized process, we next consider its physiologic relevance (Fig. 2). The prevailing thinking on the matter, based largely on animal studies, tends to treat the basolateral track as subserving “housekeeping” functions within the enterocyte during fasting: providing a wellspring of energetic substrates to tide the cell over until the next meal and preferred FA species for structural-lipid synthesis (1, 13, 15, 16, 17, 18, 68). Although up to 30% of basolateral-track FAs may be oxidized during fasting (16), FAs do not appear to be major drivers of enterocyte ATP generation in either the fed (16, 96, 97) or fasted states (16, 98) on a background of normal dietary fat content. Moreover, to the extent that enterocytes do engage in fatty acid oxidation (FAO) during energy-intensive meal absorption, most such fuel is apically derived (17).

We therefore confront a conundrum: enterocytes evidently possess robust cellular machinery for uptake and oxidation of circulating FAs, yet they seem not to use it *primarily* for energy generation, as cells typically do (16). We can envisage a few hypothetical explanations for a beefed up β-oxidative apparatus in enterocytes that encompasses a larger scale and/or a broader purpose than enterocytic energy independence during fasting (16). For example, if the enterocyte were also a “professionally” lipolytic cell, it might disburse hydrolyzed FAs locally to support other epithelial or lamina propria cells during fasting. Occurrence of such a phenomenon in vivo (e.g., calculations based on portal-drained viscera) (99) or in explanted specimens of whole intestine would not necessarily have been experimentally localizable and therefore prone to conflation with enterocyte-specific FAO. Beyond metabolism, intestinal FAO also affects epithelial proliferation and survival due to its collateral impact on the intracellular redox state (100). This may be a particularly important regulatory consideration in the tumorigenic setting of constant cell turnover and exposure to environmental toxins.

### Fat sensing

**Fig. 3:**
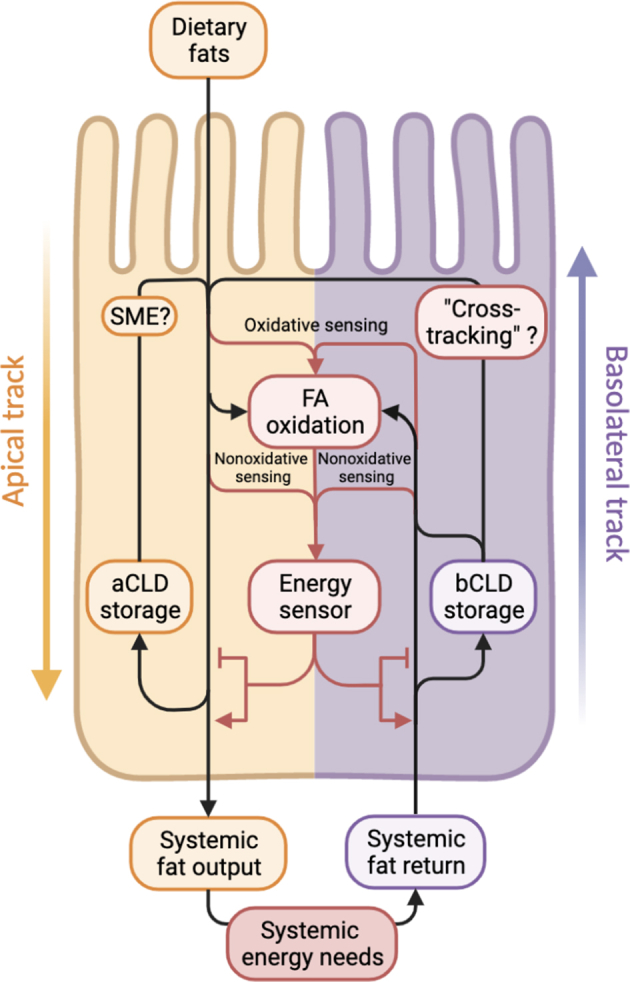
Hypothetical energy-sensing circuit, a model for interaction of apical and basolateral tracks. Fats traffic along pathways indicated by black arrows, while small quantities may be diverted along the red arrows for oxidative (i.e., via FA oxidation) or non-oxidative energy sensing to compare fat inputs and outputs as a barometer of systemic energy needs. An “energy sensor” may then control flux of fats along storage versus oxidation pathways on either track according to its homeostatic needs.

We conjecture that enterocytes harness FAO as a means of nutrient sensing (Fig. 3). Just as pancreatic β-cells co-opt glucose metabolism as a nutrient-sensing mechanism for autoregulation of insulin secretion, so may enterocytes reappropriate FA metabolism as a barometer of systemic energy balance to which they can couple their own metaboregulatory activities (101). This could occur, for example, through repurposing of FAO, which would also help to account for the enterocyte’s apparent excess capacity for FAO discussed above (16, 101). As enterocytes can take up and oxidize fat from recirculated chylomicron remnants (13, 14, 18, 19), the internal calculus of basolateral- versus apical-track FAO could constitute a fat-sensing circuit. Potentially consistent with this interpretation, augmenting enterocyte FAO in mice increases energy expenditure during high-fat diet feeding (95).

Whether through FAO or even nonoxidative means, the construction of such a fat-sensing circuit would allow the enterocyte to monitor the equilibrium between dietary (i.e., apical) fat supply and systemic demand, the latter inversely proportional to the basolaterally returning chylomicron-remnant TGs not already siphoned by other tissues. This could underpin, for example, the intestine’s observed role in feedback regulation of food intake (101). Although this concept is attractive and comports with some animal physiologic data (13, 16, 75, 102, 103, 104), particularly the independent regulation of apical versus basolateral FAO based on feeding status (16), it remains a hypothesis in need of further testing (70, 101).

Enterocyte signaling of nutrient status by any means likely requires cooperation with enteroendocrine cells, whose role in satiety and facilitation of insulin-mediated nutrient disposal is well established. For example, enterocyte nutrient-status feedback may be mediated in part via cholecystokinin production by enteroendocrine cells (105, 106). Bile acids may also play a role, given their dual functions of fat emulsification and digestion-timed signaling in multiple intestinal cell types. Bile acids reabsorbed into the circulation by ileal enterocytes go on to activate their cell-surface receptor GPBAR1 (a.k.a. TGR5) on the basolateral membranes of enteroendocrine cells (including L-cells, also enriched in the ileum) and various neurohormonal cells of the lamina propria (48, 49, 50). In this way, bile acids contribute to the human intestine’s regulated secretion of the key gut hormones GLP-1 and peptide YY (48, 49, 50, 107). Although not proven experimentally, this regulatory scheme implicates the enterocyte as well in its capacity as a dynamic gatekeeper mediating the enterohepatic circulation of bile acids (108, 109). However, the manner and extent to which these extraenterocytic processes play in enterocyte lipid metabolism remains unclear.

### FA balance

The enterocyte has access to a range of FA species from the diet that can be further strategically enriched by basolateral uptake of circulating lipoproteins and DNL (13, 86, 87). This may be important intracellularly to manage the high phospholipid throughput required for enterocytes’ constant cellular turnover (110). As FA composition governs phospholipids’ essential structural properties (111), we predict that this fundamental task requires active balancing of the cell’s FA repertoire.

Balancing the FA reserve may also be important to curate appropriately structured phospholipids that sustain lipoprotein secretion (33, 34, 111), and we speculate that ultimately it may influence the composition of the circulating FA/TG pool. In order to engage meaningfully in such a process, the basolateral track would need to influence not only the uptake of fats at the basolateral surface but also their secretion or resecretion. This process could entail some crossover of fat from the basolateral to the apical track (15, 69, 72). In so doing, basolaterally derived lipids might serve a constitutive, low-grade pump-priming function to maintain the readiness of the chylomicron assembly line and/or may even give rise to the SME (13, 14, 15, 25, 41). Cross-tracking of bCLD lipids also potentially buffers the cytoplasmic concentrations of specific FA species to optimize esterification of chylomicron-bound TGs according to enzymatic substrate preferences (40).

(Re)secretion of basolaterally derived fat could also reflect a separate basolateral lipid-secretory pathway, perhaps based on VLDL (112). Although the chylomicron is the small intestine’s canonical TRL, enterocytes are also capable of secreting apoB48-containing VLDL (i.e., distinguished operationally based on sedimentation rate), particularly during fasting (41, 113, 114, 115). This enterocyte VLDL production may occur independently of chylomicron production, and the resulting lipoproteins can differ in lipid composition (113, 114, 115, 116, 117, 118). As many lipoprotein-kinetic studies have reported effects on TRL without distinguishing between particles of differing size or density, they may inadvertently have conflated two separate processes (41).

## Enterocyte Lipid Storage in Metabolic Disease

The apparent spatial and functional separation of bCLDs from those containing newly absorbed dietary fat (aCLD) (13, 15, 16) makes it unclear a priori if bCLDs represent metabolic friend or foe. In other words, should this distinct bCLD pool be accorded the generally detrimental reputation of extraadipocellular “ectopic” lipid accumulation? Patients with chylomicron retention disease develop relatively enterocyte-specific massive apical CLD overload due to impaired intracellular trafficking of nascent chylomicrons (119, 120). However, they do not appear syndromically prone to diabetes and, interestingly, have normal serum TGs, ostensibly due to augmented hepatic DNL (119, 120). On the other hand, there are no known human disorders specifically of enterocyte bCLD metabolism. In fact, correlative human data suggest a beneficial role for enterocyte CLDs; SME magnitude, presumably reflecting the extent of prior-meal fat storage in CLD, associates positively with insulin sensitivity (25). Congruently, a mouse model of specific bCLD accumulation manifests lower plasma TG and protection from hepatic steatosis versus control, although any potential effects on glucose metabolism were not reported (13). However, reduction of CLDs by intestine-specific transgenic augmentation of lipolysis in mice does not affect TG levels (95).

If the enterocyte represents a qualified “safe haven” for short-term lipid banking, we imagine it, like adipose tissue or liver (121), will come to fail in the face of chronic fat excess and its attendant insulin-desensitizing repercussions (25). Insulin resistance and diabetes do appear to be associated with dysregulated intestinal lipid handling (69, 70, 122, 123, 124, 125, 126, 127, 128, 129, 130, 131, 132). Fat consumption produces exaggerated spikes in postprandial chylomicron-TG in patients with insulin resistance or type 2 diabetes versus healthy controls (25, 69, 122, 130, 133, 134, 135), and improved diabetes control attenuates postprandial chylomicron excursions (127, 136). Mechanistically, these findings appear to result both from increases in apoB-48 production and decreases in its clearance, as well as enhanced uptake and esterification of basolaterally (re)absorbed FFA, in the setting of insulin resistance and diabetes (69, 70, 129, 131, 135, 137, 138, 139). The finding that insulin resistance is associated with inflated postprandial TG excursions appears to conflict with the previous mentioned positive correlation between SME magnitude and insulin sensitivity. Insulin resistance thus may impair the enterocyte’s ability to siphon dietary fat for storage as CLDs during active absorption. Consequently, a greater proportion of that dietary lipid directly would enter the circulation in the prandial/postprandial period while less would remain within enterocyte CLD to resurface during the next SME.

Speculation as to the relationship between insulin resistance and dysregulated intestinal lipid metabolism calls up the question of mechanism. Although the intestine is not generally considered a classic insulin target tissue, some evidentiary support exists for a direct effect of insulin on intestinal lipid handling (89, 140). For example, insulin treatment of human fetal jejunal explants decreased the quantity of chylomicrons secreted without affecting their composition (141). On the other hand, in the setting of preexisting insulin resistance, duodenal explants from humans undergoing biliopancreatic diversion for weight control exhibited increased rates of DNL and apoB-48-TRL secretion in concert with decreased basal AKT phosphorylation versus controls (89).

Several potential mechanisms have been proposed to account for the postulated intestinal resistance to insulin. Unsuppressed FFAs themselves may produce insulin-desensitizing lipotoxic effects (121), as may their derivatives, notably ceramides (142). The small intestine of patients with the metabolic syndrome may also feature a proinflammatory milieu that, by analogy to the prevailing view in obese adipose tissue, could exacerbate insulin resistance (89). Nevertheless, we must also consider the possibility of indirect intestinal effects of insulin resistance elsewhere. Based on the previous discussion of a role for basolateral uptake of plasma FFAs in enterocyte TG synthesis, it follows that exogenous infusion of FFAs during hyperinsulinemic-euglycemic clamp prevents insulin’s suppression of apoB-48 secretion in healthy volunteers (140) but not in the chronically hyperlipidemic setting of type 2 diabetes (126). Moreover, although surgical treatment of obesity-associated insulin resistance reduced apoB-48-TRL pool size and production rate relative to the preoperative state, even in the setting of constant feeding, this may have been secondary to improvements afield, as apoB-100-TRL pool size decreased to the same extent (139).

These direct and indirect effects of insulin resistance on intestinal lipid metabolism are not mutually exclusive and may even reinforce one another. For example, insulin resistance appears to drive up the proportion of chylomicron-TG derived from recirculated (basolaterally reabsorbed) FFAs versus enteral (apically absorbed) FFAs (69). Increased intestinal TRL secretion may then provide further substrate for TG lipolysis to FFAs, including with subsequent derivatization to insulin-desensitizing ceramides (142).

Although such data implicate the intestine in the maintenance—if not also the genesis—of diabetic dyslipidemia, they do not elucidate the enterocellular processes operating between luminal fat input and chylomicron output, particularly as regards the behavior of CLD. As yet, we lack direct human evidence that insulin resistance or diabetes impacts apical or basolateral CLD physiology; circumstantial data generally support the notion but have presented interpretive difficulties. In a study of severely obese patients undergoing weight-loss surgery, TG and apoB-48 levels diverged markedly in blood (both higher) versus in jejunal explants (both lower) in patients with diabetes relative to those without it (71). These findings could suggest a role for the enterocyte as a buffer against dyslipidemia that fails in the run-up to diabetes (25). Confounding this interpretation, however, the bulk of the stained jejunal-wall neutral lipid resided in the lamina propria, likely in the form of apoB48-TRL, rather than within enterocytes proper; electron microscopic analysis was not presented (71). Surrogate measures also have not provided straightforward results. For example, small intestine specimens from insulin-resistant humans have shown both increased (89, 143) and decreased (144) expression of MTP versus control specimens. Duodenal expressions of several other genes involved in lipoprotein synthesis were lower despite *greater* apoB-48 (i.e., chylomicron) production rate and pool size in obese humans with versus without insulin resistance (144). This dissociation may result from differential impacts of hyperglycemia versus hyperinsulinemia or insulin resistance per se (145, 146). A cell-autonomous effect of hyperglycemia itself on human enterocellular lipoprotein production has yet to be demonstrated, but studies of its effects on whole-body apoB-48-TRL kinetics have yielded mixed results (126, 143, 146, 147, 148).

Finally, we once again consider bile acids (BA) given their tight correlation with insulin resistance (149, 150, 151, 152) and the antidiabetic effects of BA sequestrants (153, 154). Levels of FGF-19, a classical surrogate of intestinal BA action, are lower despite higher serum bile acid levels in insulin resistance (155, 156), although the effect of improved insulin resistance on these parameters appears to depend on the treatment modality (151, 157, 158, 159, 160, 161).

## Conclusions

We have attempted to update and streamline a dual-track model of enterocyte lipid handling, with a particular emphasis on human physiology in health and disease. Although others have also proposed elements of a dual-track model (13, 15, 16), it remains largely conceptual due to incomplete understanding of the two tracks and their relationship with one another. Both apical and basolateral tracks can silo their respective fats in distinct cytosolic lipid droplets. Both apical- and basolateral-track CLDs seem capable of participating in similar processes: FAO, structural lipid synthesis, lipoprotein (re)secretion, and storage of other lipid-soluble molecules. However, the ends for which they are employed are where the trail starts to go cold.

A central question raised by this hypothesis is the extent to which these two tracks interact. That is, do they carry out their activities purely in parallel or do they functionally intersect? We have speculated on interactions between the two tracks, including as an integrated energy-sensing circuit or as a hedge against systemic TG overload. However, these remain hypotheses in want of further testing. Key questions that remain unanswered include the precise mechanism of basolateral FAuptake (i.e., as hydrolyzed FFAs vs. as remnant TRLs), the reason for the apparent dissociation between basolateral track’s β-oxidative potential relative to its demand and why the enterocyte stores fat from each track in distinct CLD.

## Conflict of Interest

The authors have declared that no conflict of interest exists.
